# Inhibition of fibronectin accumulation suppresses TUMOR growth

**DOI:** 10.1016/j.neo.2021.06.012

**Published:** 2021-07-20

**Authors:** Hiba Ghura, Marin Keimer, Anja von Au, Norman Hackl, Verena Klemis, Inaam A. Nakchbandi

**Affiliations:** a Institute of Immunology, University of Heidelberg, Heidelberg, Germany; b Max-Planck Institute for Medical Research, Heidelberg, Germany; c Max-Planck Institute for Biochemistry, Martinsried, Germany

**Keywords:** Extracellular matrix, Fibronectin, Breast cancer, Melanoma, Pharmacologic matrix modulation, Collagen type I, pUR4, Functional upstream domain (FUD), R1R2

## Abstract

Understanding how the extracellular matrix affects cancer development constitutes an emerging research field. Fibronectin and collagen are two intriguing matrix components found in cancer. Large concentrations of fibronectin or collagen type I have been implicated in poor prognosis in patients. In a mouse model, we had shown that genetically decreasing circulating fibronectin resulted in smaller tumors. We therefore aimed to manipulate fibronectin pharmacologically and determine how cancer development is affected.

Deletion of fibronectin in human breast cancer cells (MDA-MB-231) using shRNA (knockdown: Kd) improved survival and diminished tumor burden in a model of metastatic lesions and in a model of local growth. Based on these findings, it seemed reasonable to attempt to prevent fibronectin accumulation using a bacterial derived peptide called pUR4. Treatment with this peptide for 10 days in the breast cancer local growth model or for 5 days in a melanoma skin cancer model (B16) was associated with a significant suppression of cancer growth. Treatment aimed at inhibiting collagen type I accumulation without interfering with fibronectin could not affect any changes *in vivo*.

In the absence of fibronectin, diminished cancer progression was due to inhibition of proliferation, even though changes in blood vessels were also detected. Decreased proliferation could be attributed to decreased ERK phosphorylation and diminished YAP expression.

In summary, manipulating fibronectin diminishes cancer progression, mostly by suppressing cell proliferation. This suggests that matrix modulation could be used as an adjuvant to conventional therapy as long as a decrease in fibronectin is obtained.

## Highlights

• Deletion of fibronectin in cancer cells prolongs survival
• Pharmacologic inhibition of fibronectin deposition slows down cancer progression in two models
• Decreasing fibronectin in the matrix diminishes proliferation in tumors and can serve as an adjuvant therapy

## Graphical abstract

**Figure:**
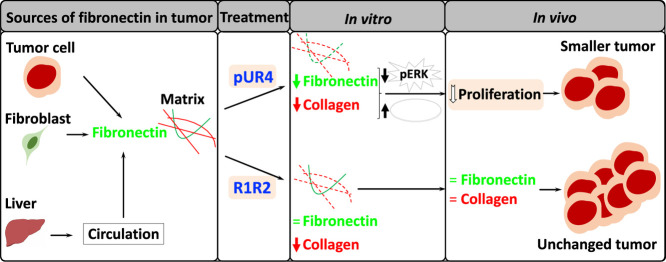

## Introduction

The extracellular matrix remains a poorly understood and undervalued player in cancer despite increasing evidence of its contribution to cancer progression [1,2]. Two members of this family of molecules of particular importance are fibronectin and collagen.

Fibronectin is a relatively large molecule found as a dimer of 240 kD each [3]. It can therefore bind to a number of cell surface receptors stimulating cell proliferation, supporting survival and promoting differentiation [1,4,5]. Fibronectin has also been evaluated in the context of cancer. In the absence of circulating fibronectin less fibronectin is incorporated in the extracellular matrix leading to a decrease in growth. Therefore, patients with higher fibronectin staining intensity in their primary tumors, reflecting increased fibronectin had a prognosis that was significantly worse than those with low fibronectin staining [1]. The reason for decreased cancer growth could be either a decrease in proliferation or/and an increase in apoptosis. Fibronectin can support proliferation by several mechanisms including by stimulating ERK phosphorylation, which translocates to the nucleus reducing sensitivity to apoptotic signals and promoting cell cycle progression [6], [7], [8]. Fibronectin also interacts with the HIPPO signaling pathway and was shown to support Yes-associated protein (Yap) translocation to the nucleus and facilitate transcription of a separate set of proliferative signals [9], [10], [11]. Apoptosis can be suppressed by fibronectin through NF-kB or activation of the PI3kinase/AKT pathway, but pFAK and Bcl-2 have also been implicated [12], [13], [14]. Another possibility is the role of fibronectin in supporting blood vessel formation [15]. In its absence during early development, embryos die due in part to failure of vessel development [16]. Early work suggested that fibronectin is required for angiogenesis [15]. Therefore, in the absence of circulating fibronectin blood vessel numbers were diminished [1]. The exact role of fibronectin in tumor angiogenesis is difficult to dissect, however, because of the contribution of various sources to fibronectin production and because of the large number of receptors to which fibronectin can bind [1,3,17].

High levels of collagen type I expression in cancer were similarly associated with poor prognosis [2]. Furthermore, the presence of fibrillar collagen increases tissue stiffness and enhances tumor progression [18]. Even though collagen has been less well studied than fibronectin, it was also found to support cell proliferation and survival [19] and inhibit apoptosis [20].

After its production and release from the cells, the matrix localizes to the intercellular space from where it binds to cell surface receptors and exerts its various functions. One possibility therefore to counter the effect of matrix is by preventing its accumulation between the cells. For fibronectin to become incorporated in the extracellular matrix its assembly in fibrils is required [21]. Through binding to mainly α5β1 integrin on the cell surface the protein is extended revealing sites needed for self-assembly [3]. One of these sites is located at the N-terminus and spans the type I domains with the numbers 1 through 9 (FN-I1–9 fragment) [22]. In contrast to fibronectin which may assemble by itself, collagen type I requires binding to the N-terminus of fibronectin in order to become incorporated in the matrix [23]. Thus, accumulation of matrix is a regulated process and requires consecutive steps.

It is possible to interfere with matrix accumulation and two molecules have been found to prevent matrix assembly. The pathogen named streptococcus pyogenes expresses fibronectin-binding domains that facilitate its invasion of epithelial cells [24]. One of these called F1 adhesin or pUR4 contains a sequence of 49 amino acids that binds to the type I domains 1 through 9 needed for fibril formation. It therefore is able to prevent fibronectin self-assembly [25]. Since this step is a prerequisite for collagen accumulation, it has been already successfully applied to prevent collagen type I accumulation in mouse models of fibrosis diminishing disease progression [26]. In contrast to pUR4, R1R2, which is produced by streptococcus equi contains two fibronectin-binding identical repeats that interact with the type I modules of fibronectin number 8 and 9. This binding prevents the interaction between fibronectin and collagen. Since this interaction is a prerequisite for fibrillar collagen accumulation collagen diminishes [23,27]. This molecule has been used in a model of lung fibrosis and seemed to diminish collagen too [28].

Our aim was therefore to investigate whether manipulation of the matrix by decreasing fibronectin and collagen accumulation in mouse models suppresses cancer growth.

## Materials and methods

### Mice

CD1 nude mice (CD1-*Foxn1nu)* were used for the MDA-MB-231 human cell injections and C57BL/6 mice were used for B16 melanoma cell injections. Mx-Cre mice were used to delete fibronectin in the circulation by mating Mx-Cre heterozygous mice with mice carrying a floxed fibronectin gene over two generations to obtain Mx-Cre_fibronectin floxed/floxed animals. Through intraperitoneal injection of polyinosinic-polycytidylic acid (pIpC; 250 µg; Amersham, Freiburg, Germany) at the age of 3 weeks administered every other day for 3 times Mx was induced resulting Cre recombinase expression and deletion of fibronectin in the cells that express Mx. Littermates not carrying Mx-Cre received pIpC similarly and were used as controls [29].

Toxicity studies were performed in C57BL/6 mice. For this, 8-week-old healthy mice received 25 mg/kg daily injections of the peptides (pUR4, R1R2 or their respective scrambled peptides) for 10 days in the toxicity experiments and in the breast cancer model and for 5 days in the B16 melanoma model. At the time of death, blood was drawn and evaluated. Animals were kept in the animal facility of the university of Heidelberg. All experiments were approved by the responsible office of the state of Baden-Wuerttemberg. Approval numbers are: G-48/08, G-139/09, G-120/11, G-245/14, G-127/15, G-1/17, G-13/17, G-34/18, G-34/19, G-85/20, G-86/20, G-187/20.

### Tumor models

The breast cancer models use a human cell line MDA-MB-231/B-luc^+^ that homes preferentially to bone and forms bone metastases. It carries a luciferase gene. Therefore, by administering luciferin, bioluminescence imaging is possible. The pictures allow estimation of the size of the tumor based on the bioluminescence signal obtained. Because it is a human cell line the recipients need to be immune compromised. CD1nu mice carrying the Foxn mutation lack mature T- cells and were used for these experiments. The melanoma skin cancer model using B16 allows inducing the cancer in immune competent C57BL/6 mice.

Intracardiac injection was performed as described [30]. Briefly, mice were anesthetized (Ketamine 120 mg/kg/xylazine and 16 mg/kg intraperitoneally in early experiments and with midazolam 5 mg/kg, medetomidine 0.5 mg/kg and fentanyl 0.05 mg/kg subcutaneously later. The antidot for the second type is: atipamezole 2 mg/kg and imidazobenzodiazepine 0.412 mg/kg). A cancer cell suspension of MDA-MB-231/B-luc^+^ selected to home to the bone marrow and establish bone metastases (10^5^/100 μl PBS) was injected in the left ventricle.

Intratibial injection was performed as described [31,32]. Briefly, mice were anesthetized as above. In addition, pain was prevented with buprenorphine 0.075mg/kg subcutaneously every 4-6 hours for three days, and two holes were drilled in the left tibia. Bone marrow was flushed out from the upper hole, which was then sealed using bone wax. MDA-MB-231/B-luc^+^ cancer cells (10^5^ per 5 μl of PBS) were injected in the lower hole using a Hamilton pipette (75RN;31/2″/3S), the hole was sealed, and the skin was sutured.

Bioluminescence imaging (BLI) was performed using IVIS-100 (Perkin-Elmer) after 5 minutes of intraperitoneal injection of D-luciferin (150 mg/kg) (Synchem, Felsberg, Germany). Dorsal and ventral measurements allowed quantification in relative light units (RLU) using the software “live imaging” (version 2.5, Xenogeny). Tumor growth was evaluated weekly starting 3 weeks after intracardiac injection by bioluminescence reporter imaging until death or euthanasia. BLI started on day 7 and up to death 40 days after intratibial injection for experiments using the knockdown cell line and for transgenic mice measurements. For animals receiving pUR4 and R1R2, BLI was performed starting day 21 and repeated on day 28. Animals were divided into comparable groups with regard to BLI and injected daily with 25 mg/kg/day of the peptides subcutaneously for 10 days.

Lytic lesions were detected by radiography using Faxitron (type 7005320, BIO-RAD). For measurement, 30 kV voltage for 3 min was used. X-rays were analyzed using Fiji/ImageJ (Wayne Rasband, NIH, Bethesda, MD). Tumors obtained at the time of death were used for histology, protein, and mRNA tests.

#### Subcutaneous melanoma model

Animals were anesthetized with Isoflurane. B16-F10 cancer cells (10^6^ per 200 μl PBS) were injected subcutaneously in the left flank. Estimation of the size at day 8 was also performed under isoflurane anesthesia. Treatment was started on day 8 for 5 days. After 13 days, tumor weight and volume (lengthxwidthxwidth/2) were determined.

### Injected peptides

The following peptides were used: pUR4 (KDQSPLAGESGETEYITEVYGNQQNPVDIDKKLPNETGFSGNMVETEDT), and its scrambled control (EKGYSKPPVGNEGGDQVDEYDTMSQTKLEDEGNTLISPITFENATEQVN), R1R2 (GLNGENQKEPEQGERGEAGPPLSGLSGNNQGRPSLPGLNGENQKEPEQGERGEAGPP) and its scrambled peptide (PGPGAEQPEQSKERNSQERGNGLALPGEELEGQEGGNKPSGENNGPPQGNLRGPLEG). Initially, the peptides contained a HIS tag (6x HIS) at the N-terminal of the molecule to allow for purification and refolding. The samples used for Fig. 5B were obtained using the HIS-tagged peptides. Later experiments were performed using purchased synthetic peptides without a HIS tag (Biocat, Heidelberg, Germany). There was no difference in growth between tagged and untagged peptides, but growth data shown are limited to the results obtained from the purchased peptides lacking a HIS tag. The peptides were administered at a dose of 25 mg/kg in 0.9% NaCl daily injected subcutaneously for 5 days in the melanoma model and 10 days in the breast cancer model.

### Cell culture

MDA-MB-231B/B-luc^±^ was cultured in Dulbecco's modified Eagle's medium (DMEM)/10% fetal calf serum (FCS) with 800 µg/ml geneticin, respectively. Cells were counted using an automated cell counter (CASY-TT; Innovatis, Mannheim, Germany). For some experiments, fibronectin-depleted FCS was obtained by running FCS on gelatin-Sepharose columns three to five times and dialysing against FCS. Successful depletion was confirmed by ELISA, and final concentration of fibronectin was 20-32 ng/ml. Knockdown of fibronectin was performed using MISSION *sh*RNA lentiviral transduction particles (Sigma #SHVR NM 002026) and transduced cells were selected based on the resulting puromycin resistance at 1 μg/ml. The control cells received an empty vector (MISSION pLKO.1-puro #SHC001V, Sigma).

B16-F10 cancer cells were cultured in DMEM/10%FCS, 100 µg/ml streptomycin, 100 IU/ml penicillin. For *in vivo* experiments cells were thawed 3-5 days before the injection and cultured for 1 day without antibiotics.

Isolation of tumor endothelial cells was performed as described [1]. Briefly, tumors were minced and digested (1 mg/ml collagenase-A (Serva, Heidelberg, Germany) + 0.05% DNAse1 (Roche, Mannheim, Germany) at 37°C for 45 minutes with shaking. Pellets were resuspended in DMEM+10% FCS. 30 μl Dynabeads (Invitrogen) were precoated with 20 µg rat anti-CD31 antibody [MEC 7.46] (Abcam) and incubated with the cells for 60 minutes at 4°C. Beads were isolated and cells separated from the beads using 0.05% trypsin/EDTA (Gibco, Invitrogen) and cultured in DMEM/20% FCS, 2 mM L-glutamine, 2 mM sodium pyruvate, 20 mM HEPES, 1% nonessential amino acids, 100 µg/ml streptomycin, 100 IU/ml penicillin, 12 U/ml fresh heparin, and 100 µg/ml EC growth supplement (Sigma). Passages 3 to 5 were used. To determine fibronectin production, cells were cultured for 72 hours in the presence of 20% fibronectin-depleted FCS.

NIH3T3 were cultured in D-MEM/10%FCS, 100 µg/ml streptomycin, 100 IU/ml penicillin. Proliferation and apoptosis of NIH3T3 cells were evaluated after 24 hours of treatment by flow cytometry where 10^5^ NIH3T3 cells were cultured in 24-well plates and treated once 10 μM (≈50 µg/ml) peptide. mRNA expression was also evaluated after 24 hours. Matrix production was evaluated after treatment for 4 days with 10 μM peptide daily. On the fifth day, cells were fixed with 1%PFA and stained for fibronectin and collagen, or lysed for western blot analysis.

### Flow cytometry

AnnexinV/PI (propidium iodide) and Ki67 stainings for determination of toxicity were performed 1 day after addition of the peptides to 10^5^ NIH3T3 cells/well. On the second day, cells were detached using cell dissociation buffer and resuspended in 50 µl of AV/PI binding buffer [10 mM HEPES (pH 7.4), 140 mM NaCl, and 2.5 mM CaCl_2_]. AV (12.5 µg/µl) was added (Alexa 647, Biolegend, #640912) for 20 minutes at room temperature in the dark, and samples were measured immediately after adding PI (1 µg/ml; Biolegend, #421301) using LSR-2 (BD Biosciences, Heidelberg, Germany). Ki67 was stained using anti-mouse Ki67-PE antibody (Biolegend, 652404, 1:50).

### Histology

Tumor sections (5 μM) were performed using a cryotome (Cryostat CM 3050, Leica). Extracellular matrix was stained after fixation in 3.7% neutral-buffered formalin using rabbit anti-mouse fibronectin (Millipore; #AB2033; 1:200); goat anti mouse collagen-type-I (Southern Biotech; #1310-01; 1:100); donkey anti-rabbit conjugated with Alexa 488 (Invitrogen; #A-21206; 1:1000); mouse anti-goat conjugated with Cy3 (Dianova; #205-165-108; 1:500). We also used rat anti-mouse CD31 (BIO-RAD; MCA2388GA; 1:100), mouse anti-alpha-smooth muscle actin (α-SMA)-Cy3 (Merck; #A2547; 1:200), rabbit anti-desmin (Dianova; #DLN-13732; 1:100), rabbit monoclonal antibody against YAP (Cell signaling #14074, 1:200), goat anti-rabbit-Alexa 647 (Abcam #ab150079; 1:1000), goat anti-mouse-Cy2 (Dianova; #115-225-166; 1:1000), goat anti-rat-Alexa 594 (Dianova; #112-585-062; 1:1000). Nuclear staining was performed using DAPI (Roth; #6335.1; 1:10000). Proliferation was evaluated by staining with an anti-mouse Ki67-Alexa 555 (BD Biosciences; 55816, 1:50). TUNEL assay was performed by serial incubation separated by washes starting with proteinase K (15 min, 1:50), 0.1% Triton-X100 (3 min), TdT-reaction buffer (5 min, ThermoFisher; #16314015), labeling reaction mix (0.25µl Biotin-11-dUTP´s; Thermo Fisher; #R0081, 2µl terminal deoxynucleotidyl transferase, TdT; Thermo Fisher; #EP0162 and 50 µl 1X TdT-reaction buffer) (2 hours), stop buffer (1.75 g NaCl and 0.88 g sodium citrate in 100 ml ddH_2_O) (5 min), staining with Avidin-Cy3 (ThermoFisher; #SA1010; 1:200) and DAPI (1:1000) for 30 min. The cells or sections were covered with Mowiol.

Slides were photographed using a ECLIPSE Ti microscope (Nikon) microscope and processed using ImageJ/FIJI (Wayne Rasband, NIH). Quantification was performed in at least three sections per mouse in at least three mice per group or more as noted in the figure legends. At least 0.9 mm^2^ was examined per section *ex vivo* or 6 mm^2^/well *in vitro*.

### RNA methods

RNA was isolated using RNAzol (Sigma; #R5433) and reversed transcribed using random primers (oligo dT primers; 25 ng/µl), RevertAid Reverse Transcriptase (ThermoFisher; #EP0442; 200 U/µl) in the presence of RiboLock RNase inhibitor (ThermoFisher; #EO0381; 40 U/µl) and dNTPs (peqlab; #20-3012; 10mM). qPCR followed using SensiFast Probe No-ROX (Bioline; #QT405-01). qPCR results were normalized to human or murine HPRT. The probes used were: collagen type I: Probe #15, human fibronectin #76, murine fibronectin #66, human HPRT #22, murine β-actin #64, murine HPRT #95 (Roche). The primers used were those suggested by Roche universal probe library.

### Protein methods

To obtain the matrix, cells were treated with 1% deoxycholate as described [33]. The insoluble fraction (DOC insoluble) represents matrix. To apply similar amounts on gel electrophoresis protein content was determined by BCA (Pierce; #23225).

For Western blotting, SDS-PAGE (12.5%) was performed and proteins were detected using the following antibodies: pERK 1/2 (cell signaling; #4376; 1:1000), ERK1/2 (cell signaling; #9102; 1:1000), pAKT Ser-473 (cell signaling; #9271; 1:1000), AKT (cell signaling; #9272; 1:1000), pFAK Tyr-397 (Cell Signaling; #8556; 1:1000), FAK (Millipore; #06-543; 1:1000), fibronectin (Millipore, #AB2033; 1:5000), collagen I (Southern Biotech; #1310-01; 1:4000), pYAP-Ser127 (cell signaling; #4911; 1:1000), YAP (cell signaling; #14074; 1:1000) and HIS tag antibody (Novagen; #70796-3; 1:1000). Detection of GAPDH was used as a loading control (Sigma-Aldrich, #G9545, 1:10,000). The secondary antibodies were as follows: goat anti-rabbit IgG-HRP, goat anti-rat IgG-HRP (Dianova; #111-035-045; 1:10000) and goat anti-mouse HRP (BioRad; #170-6516; 1:10000). Densitometry was analyzed using Fiji.

ELISA: Total fibronectin was quantified by ELISA as reported [34,35]. The antibodies used were anti-human fibronectin (Sigma-Aldrich; #3648), and mouse antibody conjugated with horseradish peroxidase (Southern Biotech; 1470-05) as described. The standard and the controls differed between human and murine ELISA. For human fibronectin the standard was isolated from outdated plasma using Gelatin-sepharose 4B and the murine standard was purchased (Dunn, #IMFBN). The samples were diluted in 1% BSA (1:2 for matrix samples and 1:4 for total lysates). Conditioned media were applied without dilution. Plasma samples were diluted 1:750, and tumor samples were diluted 1:5. Values were corrected to protein content measured by BCA (Pierce; #23225).

### Statistical methods

Analyses were performed using GraphPad Prism (version 9). Analysis of variance was performed as appropriate. If global probability was smaller than 0.05, subsequent comparisons between pairs were performed using Student's *t*, or Mann-Whitney tests as appropriate. Paired samples were evaluated using either the paired t-test or Wilcoxon matched-pairs signed rank test as appropriate. Survival was evaluated by Kaplan-Meier test. Results are expressed as mean ± SEM.

## Results

### Decreased fibronectin production leads to diminished cancer growth

Deletion of circulating fibronectin and hence diminished infiltration of fibronectin in cancer led to smaller tumors [1]. We therefore asked whether fibronectin production by the cancer cells themselves might also modulate lesion growth.

**Fig. 1:**
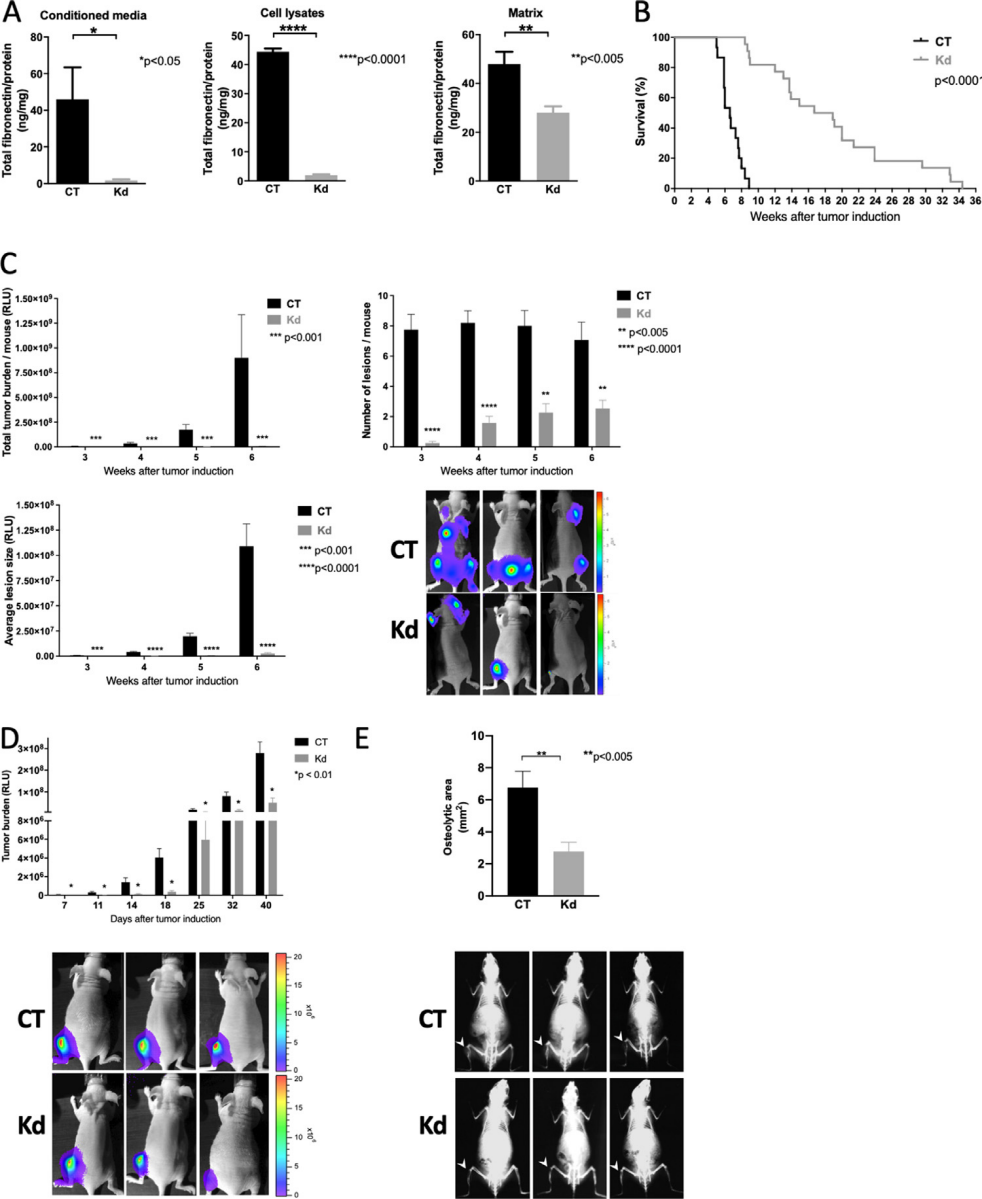
Knockdown of fibronectin diminishes cancer growth. (A) ELISA of conditioned media, cell lysates and isolated matrix (DOC-insoluble fraction) show a decrease in fibronectin in knockdown (Kd) cells compared to control (CT) cells. MDA-MB-231 cells were cultured for 3 days in the absence of fetal calf serum (FCS). Conditioned media were collected and cell lysates obtained after treatment with protein lysis buffer. Matrix was obtained after culture in the presence of 10% FCS by treating the wells with 1% sodium deoxycholate (DOC). Matrix is insoluble in DOC and can be evaluated after dissolution in SDS-containing protein lysis buffer. The number of replicates is N= 7/7 for the conditioned media, 8/6 for the cell lysates, and 20/24 for the matrix; pairs were evaluated by t-tests, *P<0.05, **P<0.005, ****P<0.0001. (B) Intracardiac injection of Kd cancer cells leads to prolonged survival compared to CT. N=38 CT/38 Kd. Survival was evaluated using Kaplan-Meier test. (C) Kd cells were associated with decreased total tumor burden after intracardiac injection of tumor cells. The number of lesions at each measurement as well as the average lesion size were also diminished. Tumor progression was evaluated using bioluminescence imaging performed weekly. Examples are shown. The number of animals in the order of the columns presented (left to right) is N=12/16, 14/19, 13/11, 11/13, with each pair representing the number of replicates for one time point. Pairs for each time point were evaluated by t-test. **P<0.005, ***P<0.001, ****P<0.0001. (D) Intratibial injection confirms a decrease in the size of Kd tumors. Breast cancer cells were injected intratibially. Growth was measured using bioluminescence imaging on days 7, 11, 14, 18 days and then weekly until euthanasia after 40 days. Examples are shown. N=25 CT/25 Kd until day 18, then 19 CT/19 Kd. Pairs were compared using t-test. *P<0.01. (E) Osteolytic lesions at day 40 were smaller in Kd tumors in the intratibial model. Examples of osteolytic lesions are shown (white arrow heads point to the lesion). N=20 CT/17 Kd. Pair compared using t-test, **P<0.005.

To do this, fibronectin was deleted in a breast cancer cell line (MDA-MB-231/B-Luc^+^). Since this is a human cell line it needs to be introduced in immune deficient mice lacking mature T-cells. This is the case in animals that carry the *Foxn1nu* mutation. The line homes preferentially to bone and carries a luciferase construct, which allows sequential evaluation of growth by bioluminescence imaging (BLI). Using shRNA we were able to diminish fibronectin production by the knockdown (Kd) cells in culture media, total cell lysates and the amount of fibronectin in the matrix only (Fig. 1A).

Injection of tumor cells into the left ventricle of mice (intracardiac model) allows the cells to be distributed throughout the body. In animals injected with Kd cells, survival was prolonged (Fig. 1 B). Improved survival was due to the suppressed total tumor burden in animals receiving the Kd cells. This in turn resulted from a lower number of lesions and smaller average lesion size (Fig. 1C), and prevented the development of complications such as paralysis of the hind limbs or weight loss which lead to death or require euthanasia of the animals. Injection of cancer cells in the tibia in a model of bone metastatic lesions (intratibial model) also confirmed a decrease in the size of the lesions as evaluated by BLI (Fig. 1D) and by quantifying the area of lysed bone at the end of the observation period of 40 days (Fig. 1E).

Thus, a decrease in fibronectin production by cancer cells inhibits cancer growth.

### Decreased fibronectin production diminishes angiogenesis and cell proliferation

**Fig. 2:**
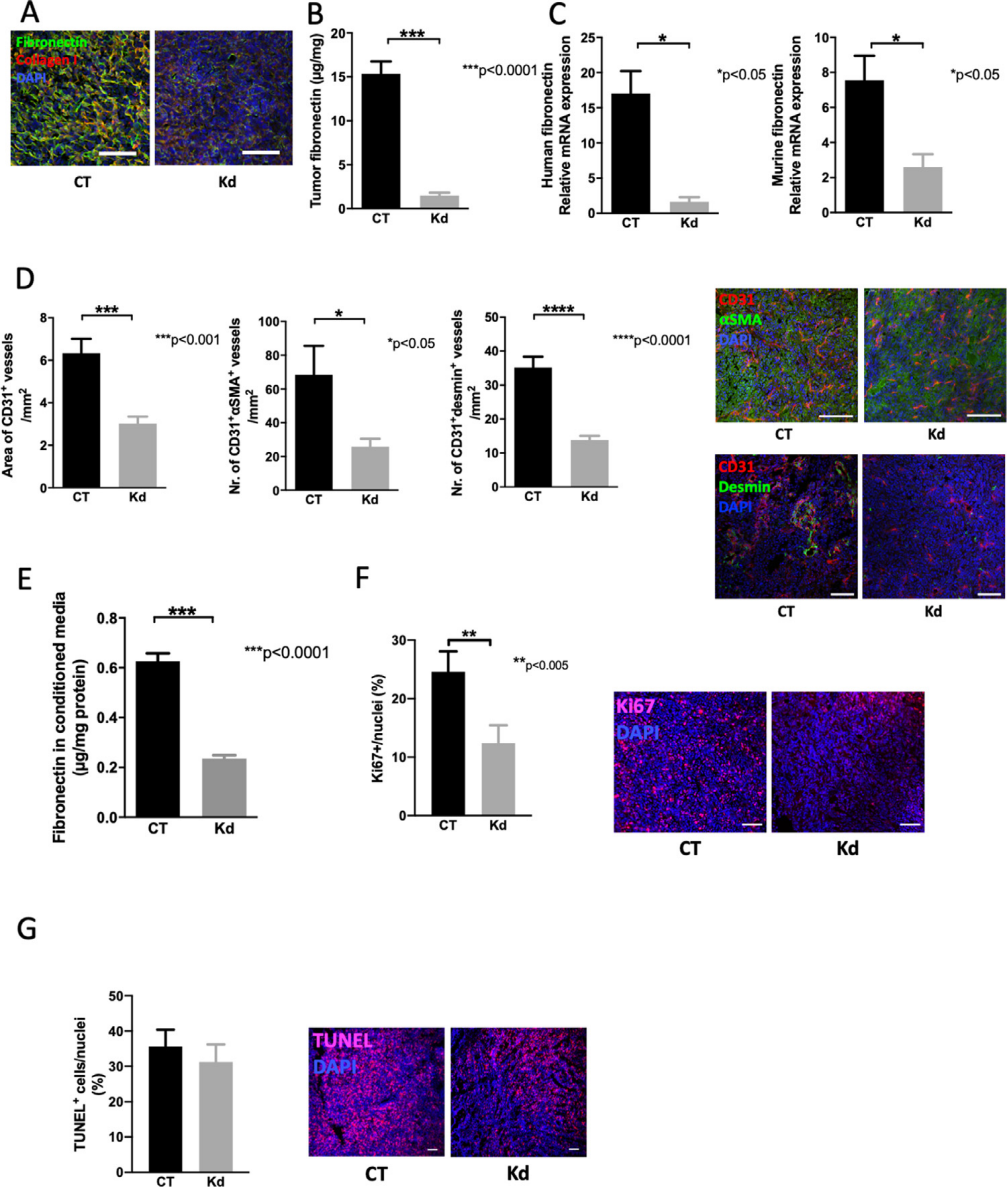
Changes in fibronectin affect blood vessels and proliferation. (A) Tumor lesions exhibit less fibronectin staining in sections from knockdown (Kd) tumors compared to controls (CT). Bars represent 100 μm. (B) Kd tumors contain less fibronectin as determined by ELISA. Pieces obtained from tumor samples were weighed and lysed in protein lysis buffer to evaluate by ELISA. The amount of fibronectin was corrected to total protein measured by the BCA method. N=3/6 replicates. (C) Expression of fibronectin mRNA originating from the cancer cells (human) and the host (murine) was diminished. Using primers specific for human and murine fibronectin it was possible to differentiate between both in qPCR analyses. Human fibronectin was corrected to human HPRT and murine fibronectin to murine β-actin. N=5/3 and 7/5 for the two graphs (left to right). (D) The area of CD31^+^ blood vessels diminishes as does the number of vessels stained with both CD31 (in red) and the pericyte markers α-smooth muscle actin (α-SMA) or desmin (in green). Examples are shown. Bars represent 100 μm. N=11/10 for CD31^+^ area and stainings with αSMA and 7/7 for stainings with desmin. (E) Endothelial cells from knockdown tumors produce less fibronectin. Isolated endothelial cells were cultured in the presence of fibronectin-depleted FCS and fibronectin evaluated in the conditioned media by ELISA. N=11/5. (F) Proliferation in tumor sections was diminished in Kd tumors as evidenced by the decrease in the percentage of Ki67^+^ cells. N=9/9. Examples are shown. Bars represent 100 μm. (G) Tumor sections were stained using TUNEL to mark apoptotic cells. No difference was seen between CT and Kd tumors. N=3/3. Examples are shown. Bars represent 100 μm. All pairs were evaluated using t-test. *P<0.05, **P<0.005, ***P<0.0001.

In order to confirm that the deletion of fibronectin in cancer cells leads to a decrease in fibronectin amount we evaluated the tumors by three methods: we stained tumor sections for fibronectin and collagen type I and saw a decrease in staining in Kd tumors (Fig. 2A). We then quantified fibronectin in tumor samples by ELISA and detected lower fibronectin amounts in Kd tumors (Fig. 2B). Finally, we measured mRNA expression for human (cancer cells) and murine (host) fibronectin and detected a drop in both in Kd tumors consistent with diminished production of fibronectin by host cells in the absence of stimulation by cancer cells (Fig. 2C).

Fibronectin supports angiogenesis [15,36]. Indeed, if deleted during embryonal development early death ensues due to impaired vasculogenesis [16]. In cancer, fibronectin has been associated with increased blood vessel formation [1]. We therefore examined angiogenesis in Kd tumors and compared it to CT tumors. We first evaluated blood vessel that are stained for CD31 only and CD31 in combination with αSMA or desmin. As shown the total area of CD31^+^ blood vessels was diminished as was the number of blood vessels that coexpress CD31 and αSMA or CD31 and desmin. This suggests less vessel formation and suppressed maturation of blood vessels (Fig. 2D). Isolated CD31^+^ cells from Kd tumors produced less fibronectin than those isolated from CT. Presumably because of the lack of stimulation by the environment (Fig. 2E).

Since fibronectin induces cell proliferation, we also stained the tumors for Ki67 and detected a significant decrease in cell proliferation in Kd sections (Fig. 2F). Evaluation of apoptosis by TUNEL staining failed to show an increase in cell apoptosis compared to CT (Fig. 2G).

In summary, a decrease in fibronectin production by cancer cells diminishes angiogenesis and proliferation. These two mechanisms cooperate to suppress cancer growth.

### Combining two reasons for decreased cancer fibronectin does not diminish cancer growth further

**Fig. 3:**
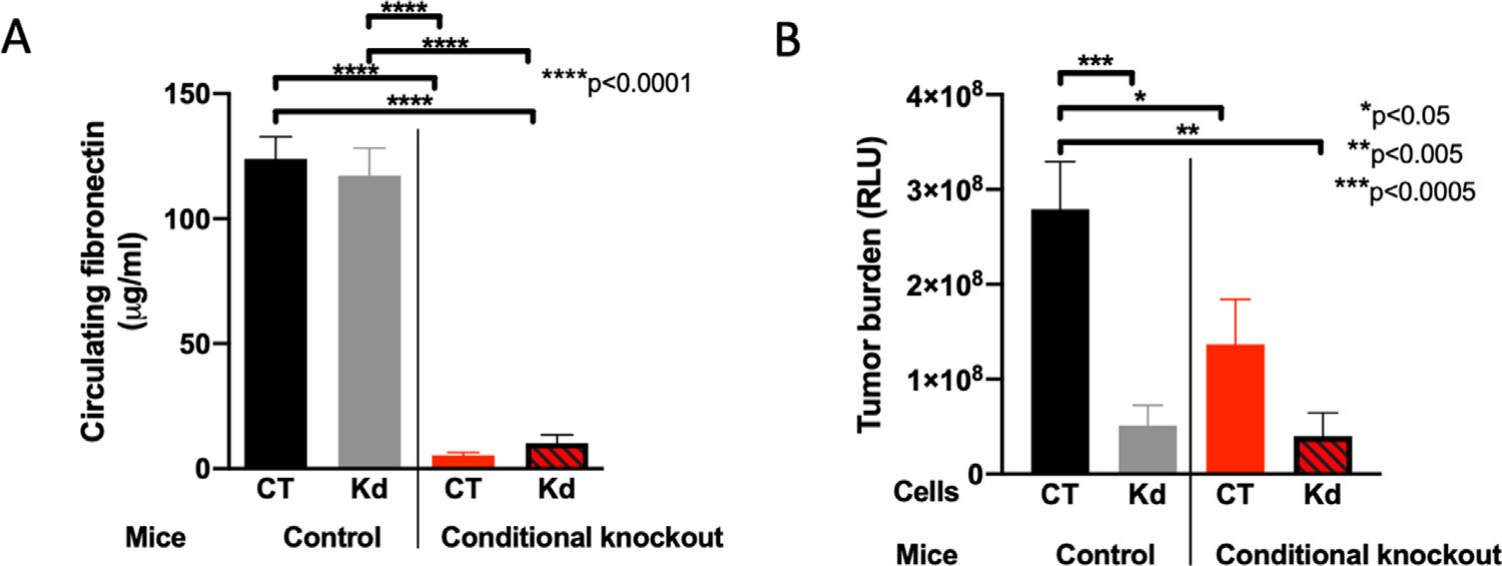
Combined deletion of fibronectin in the circulation and in cancer cells does not diminish cancer growth more than either one alone. (A) Circulating fibronectin is diminished in conditional knockout (cKO) mice lacking fibronectin in the circulation. Plasma concentration for fibronectin was evaluated prior to intratibial cancer cell injection by ELISA. N=7 CT/11 Kd mice in the control group and 9 CT/11 Kd mice in the conditional knockout group. Pairs were compared using t-test. ****P<0.0001. (B) Tumor growth of intratibial lesions is diminished to a similar degree whether fibronectin is deleted in the tumor cells (Kd in control animals) or in the circulation (CT in conditional knockout animals) or in both. Combined deletion of circulating and cancer cell fibronectin does not diminish growth more than deletion in the cancer cells or in the circulation alone. N=19/10 and 19/20 mice (the numbers represent the replicates arranged in the same order as the bars from left to right. Pairs were evaluated using t-tests. *P<0.05, **P<0.005, ***P<0.0005.

Since deletion of circulating fibronectin diminished cancer growth as published [1], and deletion of fibronectin in the cancer cells also diminished growth as shown here, we asked whether deletion of fibronectin both in the circulation and in the cancer cells will have an additive effect. We therefore used mice carrying the Cre recombinase under the control of the Mx promoter, in which both fibronectin alleles contained the floxed gene. Injection of polyinosinic polycytidylic acid (pIpC) leads to activation of the Mx promoter, expression of Cre recombinase and deletion of fibronectin (Fig. 3A). In these mice, CT and Kd cells were injected intratibially and compared to the injection in CT animals. Surprisingly, combining deletion in the circulation with deletion in the cancer cells did not diminish cancer growth further (Fig. 3B). Based on these findings we conclude that both cancer cells and the circulation contribute to fibronectin content in tumors, most likely by stimulating the production through other cell types.

Taken together, these data suggest that a decrease in fibronectin irrespective of the reason will diminish cancer growth. Therefore, manipulating fibronectin accumulation pharmacologically seems promising.

### Inhibiting fibronectin accumulation pharmacologically decreases fibronectin and collagen *in vitro* without toxicity

Since decreasing fibronectin production diminishes cancer growth, we wondered whether pharmacologic inhibition of fibronectin accumulation might also suppress tumor progression. It is possible to prevent fibronectin fibril formation with a molecule named pUR4 [24,25]. This results not only in decreased accumulation of fibronectin but also lowers collagen, because fibronectin fibrils are needed for collagen incorporation in the matrix [27,37]. Another molecule called R1R2 prevents attachment of collagen upon fibronectin fibrils, but with no measurable effect on fibronectin itself [23].

**Fig. 4:**
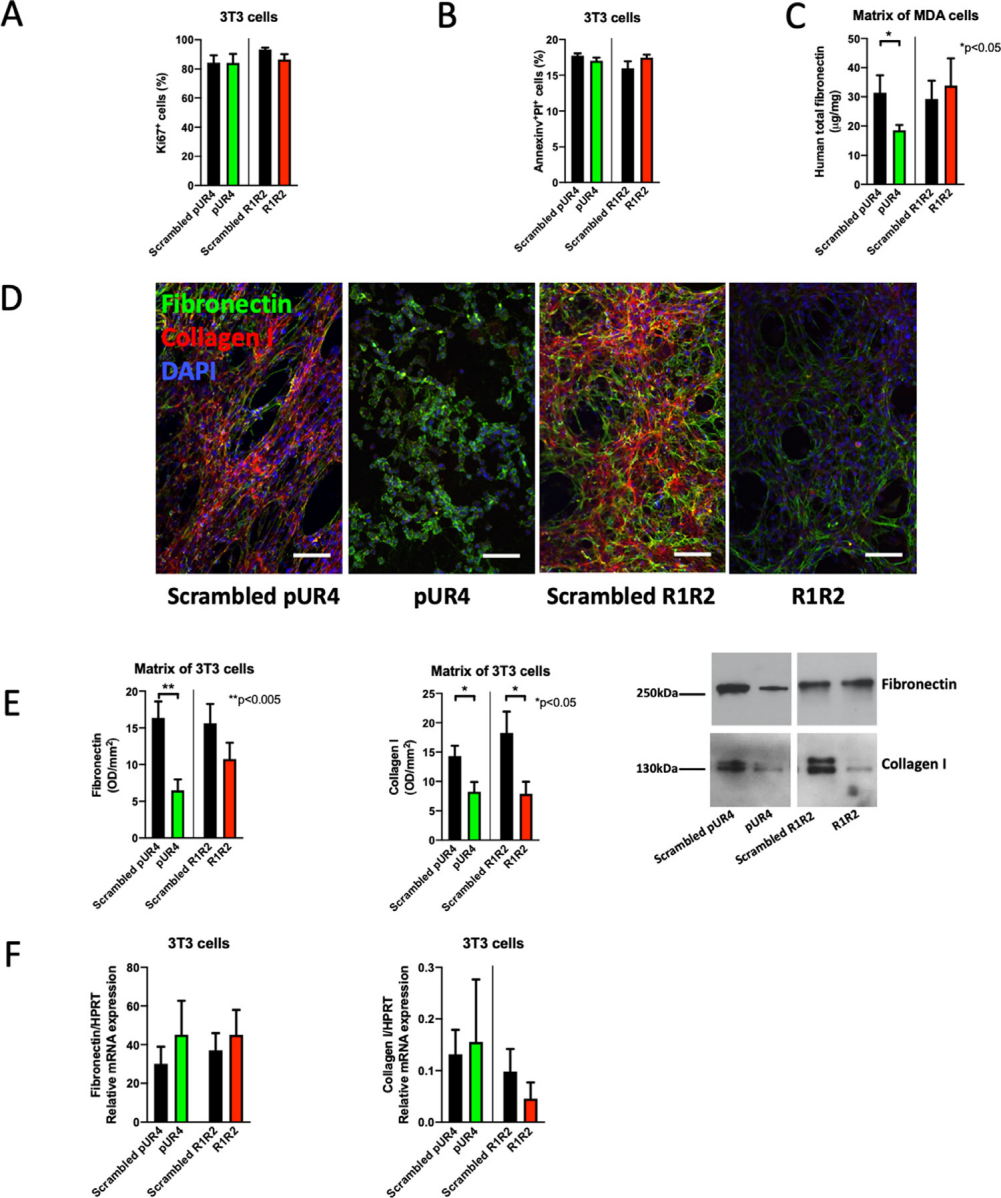
*In vitro* effects of pUR4 and R1R2. (A) Neither pUR4 nor R1R2 affect cell proliferation as evidenced by Ki67 staining after addition of 10 μM of either peptide and evaluating proliferation after 24 hours by flow cytometry. N=5/5/5/5 replicates for each treatment. (B) Similarly, neither pUR4 nor R1R2 increase apoptosis as evidenced by AnnexinV^+^PI^+^ by flow cytometry after addition of 10 μM of either. N=4/4/4/4 replicates for each treatment. No differences were detected by t-tests in A or B. (C) pUR4 lowers fibronectin in the matrix of MDA-cancer cells. Matrix (DOC-insoluble fraction) was isolated from cancer cell cultures using MDA-MB-231/B-luc+ for 4 days in the presence of FCS, treated daily with the peptides (10 μM) and evaluated by ELISA. The amount was corrected to total protein measured by the BCA method. N=17/22/12/14 replicates (arranged in the same order as the bars). Comparison by t-test. *P<0.05. (D) Staining of fibronectin (green) and collagen (red) in cultures of 3T3 fibroblasts maintained for 4 days in FCS-containing medium and with daily addition of the peptides shows that pUR4 completely prevented fibronectin matrix assembly. The green seen is intracellular fibronectin. Collagen type I was absent in both pUR4 and R1R2 treated fibroblasts. Bars represent 100 μm. Note that the peptides were added within 1 hour after culturing the cells. (E) The same experiment was performed as in D, but the matrix was obtained (DOC-insoluble fraction) and evaluated by Western blotting for fibronectin and collagen I. The amount of protein was measured by BCA and equivalent protein amounts were applied to the gel, because GAPDH cannot be detected in the DOC-insoluble fraction. Fibronectin diminished only with pUR4 treatment, while collagen I diminished with both treatments. N=15/15/7/7 replicates. (F) Neither pUR4 nor R1R2 modulate mRNA expression of fibronectin and collagen I as measured by qPCR. 3T3 fibroblasts were treated once with 10 μM of the peptides and mRNA isolated 24 hours later. N=12/11/11/11 and 14/11/15/12 replicates (The numbers represent the replicates for each treatment and are arranged in the same order as the bars from left to right).

We first evaluated whether pUR4 or R1R2 affected proliferation and apoptosis *in vitro*. Exposing NIH3T3 cells to both molecules for 24 hours did not show any effect. This excludes direct toxicity by pUR4 or R1R2 on the cells (Fig. 4A-B).

Cancer cells that produce normal amounts of fibronectin were treated with pUR4 or R1R2 daily for 4 days, followed by evaluation of fibronectin in the matrix. pUR4 lowered fibronectin by ELISA (Fig. 4C). Similarly, treating 3T3 fibroblasts with pUR4 or R1R2 suppressed fibronectin and collagen matrix accumulation using pUR4. R1R2, on the other hand, only diminished collagen matrix accumulation (Fig. 4D). Western blotting of the matrix confirmed a decrease in fibronectin after pUR4 treatment and in collagen after treatment with either pUR4 or R1R2 (Fig. 4E). This decrease was not due to a change in fibronectin or collagen type I mRNA expression (Fig. 4F).

Taken together, these data suggest that pUR4 is able to diminish both fibronectin and collagen accumulation in the matrix.

### Inhibiting fibronectin accumulation pharmacologically suppresses cancer growth in two models

**Fig. 5:**
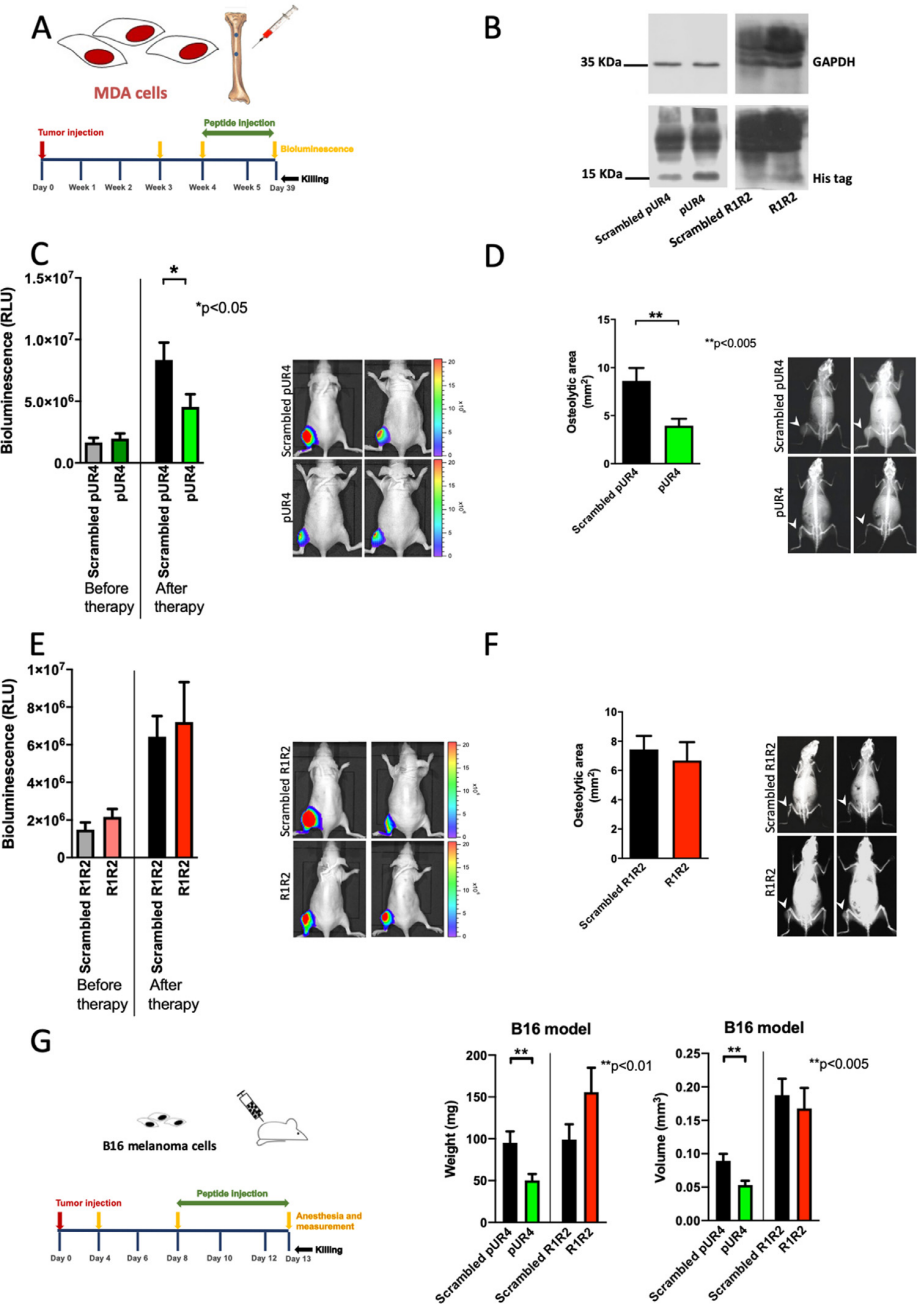
pUR4 diminishes cancer growth but R1R2 does not. (A) Immune deficient mice were injected with MDA-MB-231/B-luc^+^ intratibially and growth evaluated after 21 and 28 days. Based on bioluminescence imaging results, the mice were divided into comparable groups and on day 29, treatment with the molecules was started. Each mouse received 25 mg/kg/day for 10 days. At the end of the experiment on day 39, bioluminescence measurement was repeated, an x-ray performed and the mice euthanized. Results for these experiments are shown in (B-F). (B) pUR4, R1R2 and their respective scrambled controls contained a HIS tag. The tumors were induced by intratibial injection and the peptides injected daily starting on day 29 until euthanasia on day 39. The tag was detected in tumor tissue by Western blotting after daily injection of the peptides for 10 days confirming that they reach the tumor. (C-D) Breast cancer cells were injected intratibially and growth evaluated by bioluminescence imaging. At day 28 (before therapy) the mice were divided in two groups that were treated with scrambled pUR4 or pUR4. The peptides were administered daily by subcutaneous injections starting on day 29 for 10 days at a dose of 25 mg/kg/day. (C) Bioluminescence measurements after therapy showed that treatment with pUR4 decreased growth compared to scrambled pUR4. N=17/20. (D) X-ray analysis of the tibia revealed a decrease in osteolytic area consistent with the decrease in bioluminescence signal after pUR4 therapy (white arrow heads point to an osteolytic lesion). N=7/17. Pairs were compared by t-test. *P<0.05, **P<0.005. (E-F) Breast cancer cells were injected intratibially and growth evaluated by bioluminescence imaging. At day 28 (before therapy) the mice were divided in two groups that were treated with scrambled R1R2 or R1R2. The peptides were administered daily by subcutaneous injections starting on day 29 for 10 days at a dose of 25 mg/kg/day. (E) Bioluminescence imaging at the end of the experiment on day 39 confirmed that growth was not affected by R1R2 treatment after therapy. N=34/29. (F) Evaluation of osteolytic lesions at the end of the experiment also did not show a significant difference between the scrambled peptide and R1R2 (white arrow heads point to the osteolytic lesion). (G) On day 8 after injection of 10^6^ B16 cells subcutaneously volume was estimated and mice divided in comparable groups. Injection with the peptides was started on the same day with 25 mg/kg/day for 5 days. On day 13 animals were euthanized. pUR4 diminished growth but R1R2 did not in a model of B16 subcutaneous melanoma cells as evaluated by weight and volume. N=51/51/19/22 replicates (The numbers represent the number of treated mice arranged in the same order as the bars). Pairs were compared by t-tests. **P<0.01 for weight and **P<0.005 for volume.

Using the breast cancer model of bone metastatic lesions, we sought to evaluate how matrix modulation might affect growth of these tumors. Induction of intratibial lesions was followed by bioluminescence measurements to determine size. Mice were divided in groups with comparable mean signal (RLU: relative light units) and comparable range for each pair. Starting on day 29, pUR4 or R1R2 were injected subcutaneously daily at 25 mg/kg/day for 10 days followed by repeat bioluminescence measurement and x-ray evaluation of the osteolytic lesion in the tibia after 10 days of treatment (Figure 5A).

Since the peptides had a HIS-tag attached, it was possible to confirm that both peptides reached the tumor (Figure 5B). As shown, treatment with pUR4 resulted in a weaker bioluminescence signals and smaller osteolytic lesions in the tibia (Figure 5C-D). In contrast, R1R2 did not affect growth as defined by bioluminescence imaging or the size of osteolytic lesions (Fig. 5E-F).

In order to determine whether this effect was limited to epithelial tumors exemplified by the breast cancer cells, we evaluated whether the two molecules pUR4 and R1R2 had any effect on the growth of the murine melanoma B16 cells administered subcutaneously. Injection of 10^6^ melanoma cells in one flank subcutaneously was followed on day 8 by measurement of the tumor. Estimation of the volume allowed distribution of the animals in 4 comparable groups that were then treated with the peptides pUR4 and R1R2 at a dose of 25 mg/kg/day for 5 days. At the time of death, weight and volume of the tumor were determined as a surrogate for cancer progression. Similarly, to the breast cancer model, pUR4 was able to suppress B16 tumor volume and weight, while R1R2 was not (Fig. 5G).

Thus, in two experimental cancer models, pUR4 diminishes cancer growth while R1R2 fails to affect the tumors.

### Decreasing fibronectin content diminishes proliferation

**Fig. 6:**
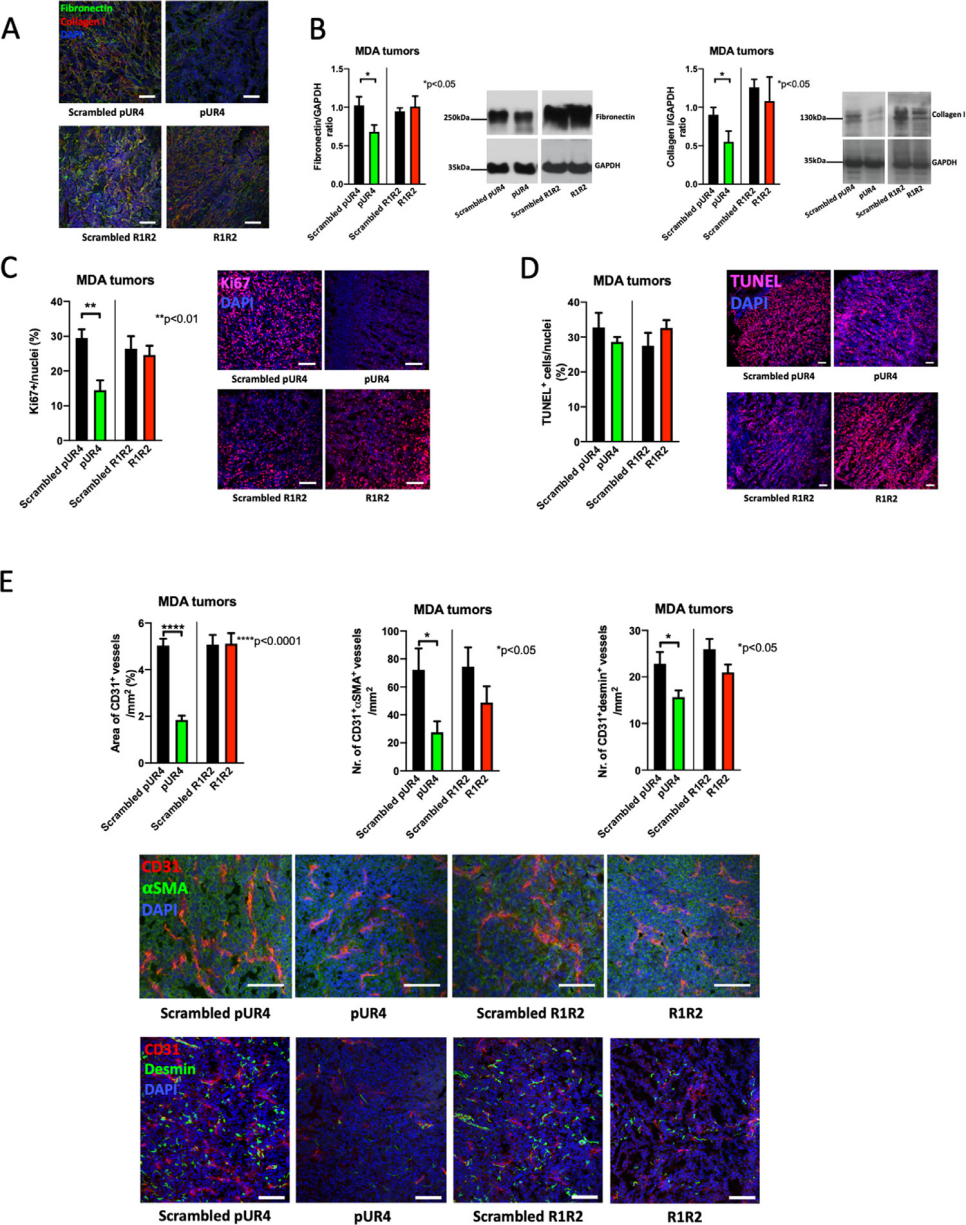
Histology of pUR4-treated tumors confirms changes in matrix and reveals a decrease in proliferation. (A) Stained tumor sections show a decrease in fibronectin and collagen in pUR4-treated animals and no changes in R1R2-treated animals. Bars represent 100 μm. (B) Western blots from the tumors confirm a decrease in fibronectin and collagen with pUR4 treatment, but collagen I did not decrease with R1R2 therapy. N=4/4/4/4 replicates/treatment. Relevant pairs were evaluated by t-tests. *P<0.05. (C) Proliferation was diminished after treatment with pUR4. Sections were stained for Ki67. Bars represent 100 μm. N=9/9/9/9. Pairs were evaluated by t-tests. **P<0.01. (D) Apoptosis was not affected *in vivo* by the various treatments. Sections were stained for TUNEL. Examples are shown. Bars represent 100 μm. N=9/9/9/9. Pairs were evaluated by t-tests. (E) Evaluation of blood vessels shows a decrease in area of CD31^+^ vessels with pUR4 treatment as well as the number of CD31^+^αSMA^+^ or CD31^+^desmin^+^ double positive vessels (adjusted to the area). Sections from representative tumors with bars representing 100μm. These are intratibial tumors from mice treated for 10 days with the peptides. N=12/12/11/12 for CD31 alone or with α-smooth muscle actin (αSMA) and 11/10/7/7 for staining with desmin. Pairs were compared by t-test. *P<0.05, ****P<0.0001.

In order to determine the reason for decreased growth with pUR4 but not with R1R2, we evaluated both fibronectin and collagen in MDA-MB-231 tumors. Staining of tumors from mice treated with pUR4 showed a decrease in both fibronectin and collagen (Fig. 6A) that was confirmed by western blots (Fig. 6B), while staining of tumors from mice treated with R1R2 or Western blots failed to show a decrease in collagen I (Fig. 6A-B).

Since fibronectin stimulates proliferation, we also evaluated proliferation (by Ki67 staining) and apoptosis (by TUNEL staining) in these cancers. Proliferation was diminished in pUR4-treated tumors from the breast cancer model (Fig. 6C). In contrast, no change in apoptosis was detected (Fig. 6D). R1R2 affected neither proliferation nor apoptosis (Fig. 6C-D).

Evaluation of the blood vessels showed that, similarly to Kd tumors, pUR4 was associated with a decrease in the total area of CD31^+^ vessels, as well as the number of vessels that express both CD31 and αSMA or both CD31 and desmin (Fig. 6E).

In summary, pUR4 suppresses cancer growth by inhibiting proliferation and ultimately decreasing new blood vessel formation.

### ERK and YAP phosphorylation are affected by pUR4

**Fig. 7:**
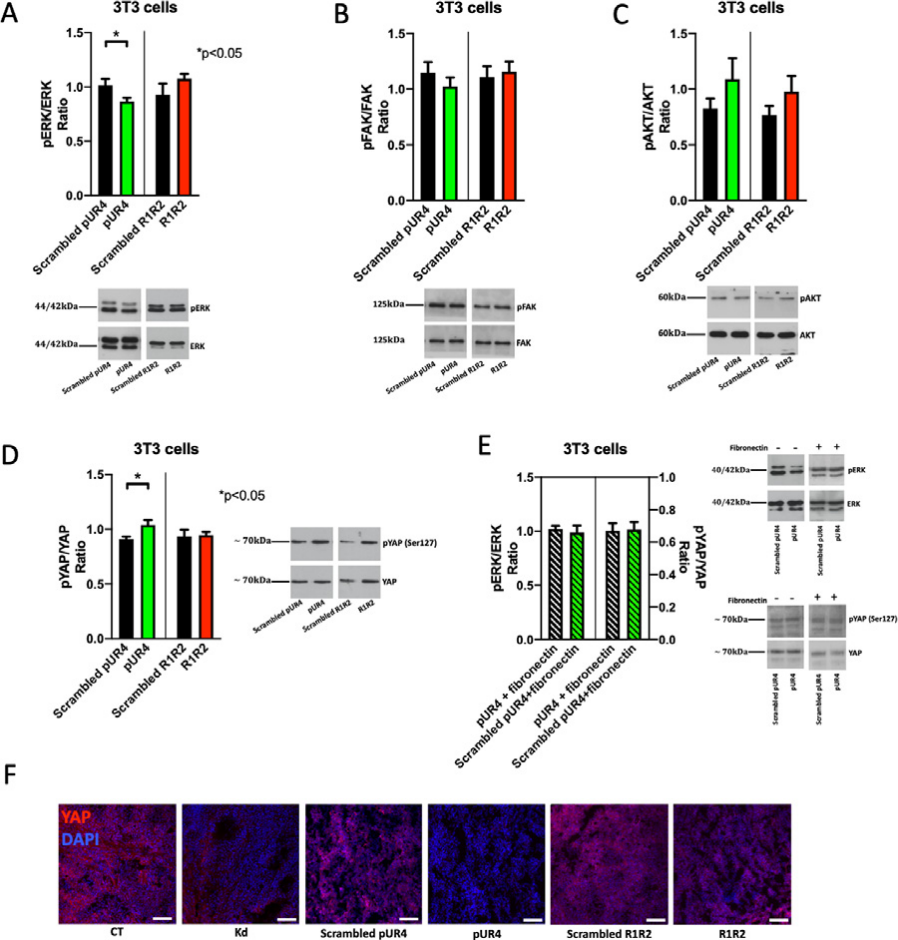
Intracellular signaling is affected by the presence of pUR4. (A-C) Exposure of 3T3 cells to pUR4 is able in the short term to decrease ERK phosphorylation without affecting FAK or AKT phosphorylation. 3T3 were cultured overnight in the absence of FCS and then treated for 15 minutes with 10 μM of the peptides. N=14/14/12/12 (from left to right) for ERK, 14/14/14/14 for FAK, 12/12/10/10 for AKT. Relevant pairs were compared by t-tests. *P<0.05. (D) Exposure of 3T3 cells to pUR4 is able in the short term to increase YAP phosphorylation. 3T3 were cultured overnight in the absence of FCS and then treated for 45 minutes with 10 μM of the peptides. N=8/8/8/8. Pairs were compared by t-tests. *P<0.05. (E) When fibronectin is administered together with pUR4 there is neither a decrease in pERK nor an increase in pYAP. 3T3 were cultured overnight in the absence of FCS and then treated for 15 minutes with 10 μM of the peptides with fibronectin 500 ng/ml. N=4/4 for pERK/ERK and 4/4 for pYAP/YAP. (F) YAP staining in the tumors suggests a decrease in total YAP consistent with decreased proliferation and angiogenesis in Kd and in pUR4-treated tumors.

Fibronectin usually binds to integrins and is able to increase proliferation by stimulating ERK phosphorylation [7]. This increase in proliferation contributes to cancer progression [38]. In order to determine why proliferation was diminished in tumors exposed to pUR4 we treated 3T3 cells for 15 minutes with pUR4 and were able to confirm a decrease in pERK/ERK. One possible explanation is that in the presence of pUR4, baseline stimulation by fibronectin is impaired. Neither pFAK nor pAKT were affected, however (Fig. 7A-C).

It has also been reported that fibronectin regulates HIPPO signaling [9], which both stimulates proliferation and angiogenesis [39], [40], [41]. The yes-associated protein (YAP) is part of the HIPPO pathway. Activation of HIPPO increases phosphorylation of YAP leading to its degradation. Less YAP translocates to the nucleus and proliferation is diminished [41,42]. Therefore, more pYAP and less YAP is detrimental to cancer [43]. We therefore asked whether pUR4 exposure by preventing interaction with fibronectin might affect YAP phosphorylation. Treatment of 3T3 cells with pUR4 resulted after 45 minutes in an increase in pYAP, an effect that is consistent with decreased proliferation (Fig. 7D).

Both the change in pERK and in pYAP were due to lack of fibronectin interaction with the cell in the presence of pUR4, because co-administering fibronectin with pUR4 normalized both pERK/ERK and pYAP/YAP (Fig. 7E). More importantly, the increase in pYAP in the presence of pUR4 should lead to diminished total YAP. Indeed, YAP expression was decreased in Kd tumors and in tumors exposed to pUR4 as suggested by staining (Fig. 7F).

In summary, pUR4 administration interferes with fibronectin-mediated signaling providing an explanation for decreased proliferation.

## Discussion

**Fig. 8:**
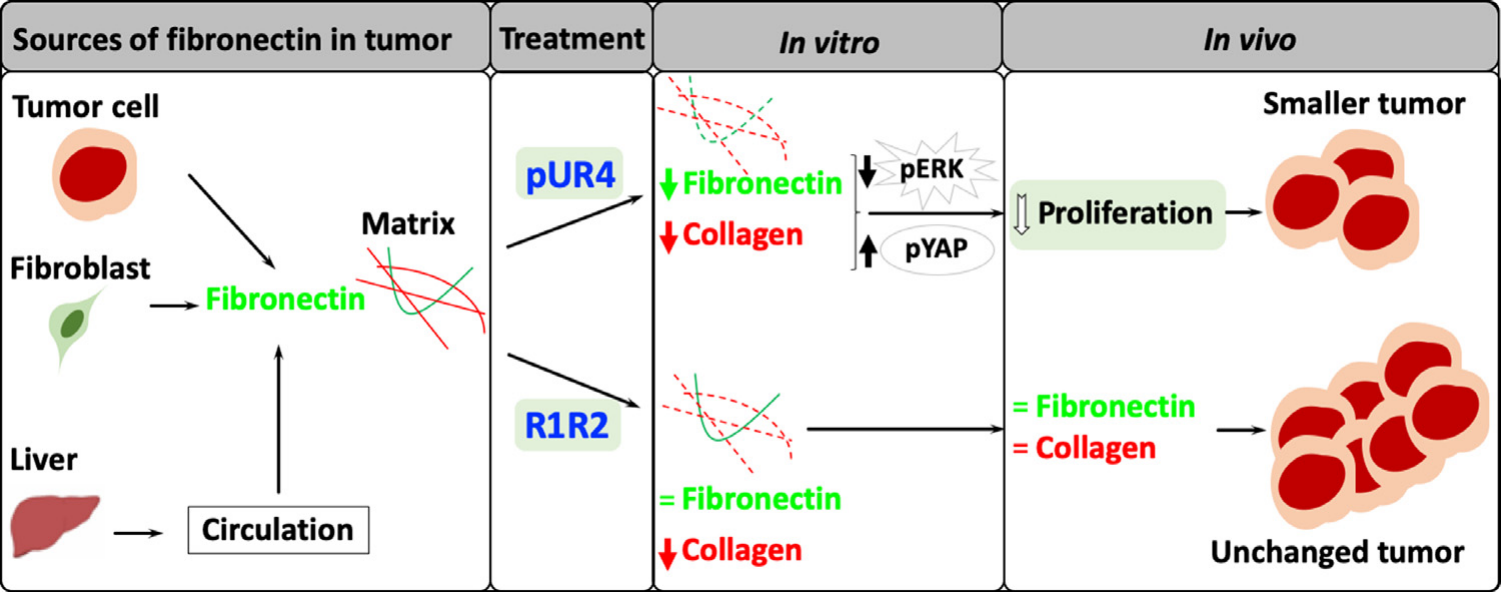
Summary. Fibronectin produced by tumor cells, fibroblasts, and coming from the liver through the circulation contributes to matrix in tumors. Treatment with pUR4 diminishes fibronectin and collagen type I leading to inhibition of ERK activation and stimulation of YAP degradation (resulting from increased pYAP). This eventually suppresses proliferation and results in smaller tumors. Treatment with R1R2 did not affect fibronectin but diminished collagen type I *in vitro*. It did not however change collagen I *in vivo* nor did it affect tumor size. Thus, manipulating fibronectin with pUR4 slows down cancer progression.

The current study shows that pharmacologic manipulation of fibronectin is possible and beneficial in two experimental models of cancer. Interfering with Fibronectin (and collagen) accumulation using pUR4 led to smaller tumors as evaluated by bioluminescence imaging (BLI) and osteolytic area on x-rays in the breast cancer model and as evaluated by weight and volume in the melanoma model. This is most likely the result of lower fibronectin and consequently suppressed proliferation *in vivo* (Fig. 8).

Even though R1R2 should have decreased collagen type I in the tumor by changing the availability of the collagen binding site on fibronectin [3,23], it did not by Western blotting. The detection of the histidine (HIS) tag in the tumors of injected animals suggests that the molecule was able to infiltrate the tumor. It is possible that the amount that reached the tumor was not enough, but this seems unlikely, because the same dose was used for both pUR4 and R1R2, both molecules are relatively small and pUR4 succeeded in affecting the matrix. R1R2 was able to diminish collagen I accumulation in a model of lung fibrosis when administered intratracheally [28], and this was attributed to changes in myofibroblast differentiation. In our hands, however, R1R2 failed to affect mRNA expression of fibronectin or collagen I or significantly diminish αSMA^+^ or desmin^+^ blood vessels (Fig. 6E). It is also possible that R1R2 is unable to bind and mask the relevant collagen binding site on fibronectin *in vivo* in tumors because of differences in matrix composition between fibrosis and tumor. The decrease in collagen type I in pUR4-treated animals therefore suggests that the main mechanism for decreasing collagen I in tumor would be by minimizing the availability of fibronectin.

Suppressed tumor growth by pUR4 treatment is associated with diminished proliferation, some changes in angiogenesis and no effect on apoptosis. Since pUR4 by itself did not affect proliferation *in vitro*, this must therefore be an indirect effect mediated by changes induced by pUR4 in the tumor. Two matrix proteins were changed by pUR4 treatment, fibronectin and collagen. The decrease in proliferation is similar to that seen in knockdown tumors lacking fibronectin, and suggests that this is, at least in part, mediated by the decrease in fibronectin. Indeed, fibronectin stimulates proliferation by several mechanisms. Fibronectin binding to α5β1 integrin leads to phosphorylation and hence activation of extracellular-signal related kinase (ERK) [7]. This is followed by translocation to the nucleus, a tightly regulated step associated with proliferative response [44]. Attachment of the cells to fibronectin fibrils also suppresses p38 activity. A crosstalk between ERK and p38 takes place, and both ERK activation and p38 inhibition stimulate proliferation [45]. The YES-associated protein (YAP) of the HIPPO tumor suppressor pathway was also shown to regulate cell proliferation [46]. Overexpression of YAP for example enhanced proliferation, while a decrease was associated with better prognosis [47,48]. Since more phosphorylation of YAP would hasten its degradation, and concomitantly inhibit proliferation, the decrease in proliferation seen with pUR4 treatment could therefore be due to the combination of suppressed ERK phosphorylation and enhanced YAP phosphorylation. Inhibiting cancer growth by a combination of decreasing ERK phosphorylation and counteracting the HIPPO signaling pathway has been discussed as a possibility for improving therapy outcome and prognosis in cancer [49,50]. Based on the current findings, this is achieved by decreasing fibronectin accumulation with pUR4, suggesting that such an intervention might support other therapies against cancer without major complications.

Fibronectin regulates anoikis, the programmed cell death that results from failed attachment to the matrix [51]. It was therefore surprising that both in Kd tumors and in pUR4-treated animals there was no change in apoptosis despite the decrease in fibronectin (Figs. 2A-B and 6A-B). It is possible the remaining fibronectin or the other matrix proteins were enough to prevent an increase in apoptosis. The data nevertheless suggest that pUR4 does not exert its effect by enhancing programmed cell death.

The findings of blood vessel evaluation may seem unexpected at first, but can be explained based on the literature. Deletion of fibronectin during embryonal life leads to early death shortly after implantation in part because of developmental vascular defects [16]. Fibronectin has also been implicated in cancer angiogenesis [1]. It was therefore expected that pUR4 by decreasing fibronectin will diminish blood vessel formation, possibly to a milder degree than in fibronectin Kd tumors. The decrease in the number of vessels that co-express αSMA or desmin together with CD31 cannot be explained by a drop in the isoform containing EDA, which had been thought to modulate fibroblast activation, because its deletion did not affect the number of αSMA cells around the newly formed blood vessels [52]. Therefore, the decrease in pericyte marker expressing cells is not due to a direct effect of fibronectin, but might result from a change in matrix composition. Since blood vessels that lack pericytes in their walls are less stable, the vessel area is diminished [53]. It is also possible that collagen drop contributed to suppressing vessel formation [54]. Since YAP from the HIPPO pathway additionally stimulates angiogenesis [39,40], lower YAP (Fig. 6E) might further contribute to the decrease in angiogenesis seen in Kd and pUR4-treated tumors. Thus, pUR4 diminished angiogenesis due to less available fibronectin, but the change in collagen could have contributed to the changes seen in Fig. 6E.

In this work we only evaluated the effect of pUR4 and R1R2 on tumor size. In the model of local breast cancer lesion we found an effect of pUR4 on diminishing bioluminescence signal reflecting luciferase content in tumor cells, and this was confirmed by evaluating the size of osteolytic bone lesions induced by the tumor. In the case of the B16 melanoma model we only evaluated the weight and the volume of the primary lesion. The decrease in proliferation after treatment with pUR4 is consistent with decreased growth. This work, however, does not address whether pUR4 might affect the development of metastatic disease. This is particularly relevant because fibronectin influences the availability of various growth factors such as VEGF and TGF-β that modulate tumor growth [1,55]. It also supports chemotaxis [56,57]. Changing the availability of fibronectin in the tumor pharmacologically by decreasing its content might therefore affect migration of the cells out of the tumor lesion and consequently the development of distant metastatic lesions. Since fibronectin represents part of the premetastatic niche [58], it is also possible that decreasing its accumulation (and the stored growth factors) might affect the composition of the niche making it less hospitable and diminishing the development of metastatic lesions. Since deletion of fibronectin in tumor cells diminished the number of metastatic lesions in the intracardiac model (Fig. 1C), it is possible that pUR4 also exerts beneficial effects on the development of metastatic disease. This will need to be evaluated in an adequate metastasis model.

Decreasing fibronectin in cancer irrespective of how this is achieved, by deletion of fibronectin in cancer cells, by decreasing circulating and hence infiltrating fibronectin or by preventing its accumulation by treatment with pUR4 diminishes cancer growth. Of note is that the cancer does not shrink in size with exposure to pUR4. This treatment only slows down progression (Fig. 5C and 5E) by suppressing proliferation and possibly indirectly affecting angiogenesis. It is therefore tempting to speculate that manipulating matrix using pUR4 might be useful as an adjuvant in the treatment of cancer.

## Authors contributions

IAN, HG: Conceptualization; HG, MK, AvA, NH, VK: Data curation and formal analysis; IAN: Funding acquisition; HG, MK, AvA, NH: Investigation; HG, MK, AvA, NH, VK: Methodology; IAN: Project administration; IAN: Resources; IAN: Supervision; HG, AvA, NH: Validation; HG, MK and IAN: Visualization: IAN and HG: Writing - original draft and Writing - review & editing.
